# Differential gene expression analysis of *in vitro *duck hepatitis B virus infected primary duck hepatocyte cultures

**DOI:** 10.1186/1743-422X-8-363

**Published:** 2011-07-23

**Authors:** Sajith Nair, Devaki S Arathy, Aneesh Issac, Easwaran Sreekumar

**Affiliations:** 1 Molecular Virology Laboratory, Rajiv Gandhi Centre for Biotechnology (RGCB), Thycaud P.O., Thiruvananthapuram-695014, Kerala, India

## Abstract

**Background:**

The human hepatitis B virus (HBV), a member of the hepadna viridae, causes acute or chronic hepatitis B, and hepatocellular carcinoma (HCC). The duck hepatitis B virus (DHBV) infection, a dependable and reproducible model for hepadna viral studies, does not result in HCC unlike chronic HBV infection. Information on differential gene expression in DHBV infection might help to compare corresponding changes during HBV infection, and to delineate the reasons for this difference.

**Findings:**

A subtractive hybridization cDNA library screening of *in vitro *DHBV infected, cultured primary duck hepatocytes (PDH) identified cDNAs of 42 up-regulated and 36 down-regulated genes coding for proteins associated with signal transduction, cellular respiration, transcription, translation, ubiquitin/proteasome pathway, apoptosis, and membrane and cytoskeletal organization. Those coding for both novel as well as previously reported proteins in HBV/DHBV infection were present in the library. An inverse modulation of the cDNAs of ten proteins, reported to play role in human HCC, such as that of Y-box binding protein1, Platelet-activating factor acetylhydrolase isoform 1B, ribosomal protein L35a, Ferritin, α-enolase, Acid α-glucosidase and Caspase 3, copper-zinc superoxide dismutase (CuZnSOD), Filamin and Pyruvate dehydrogenase, was also observed in this *in vitro *study.

**Conclusions:**

The present study identified cDNAs of a number of genes that are differentially modulated in *in vitro *DHBV infection of primary duck hepatocytes. Further correlation of this differential gene expression in *in vivo *infection models would be valuable to understand the little known aspects of the hepadnavirus biology.

## Introduction

The human hepatitis B virus (HBV) and the duck hepatitis B virus (DHBV), which are members of the same virus family, hepadnaviridae, share several features in common [1]. Unavailability of primary animal models susceptible to HBV infection, and inefficiency and unreliability of the infection process in *in vitro *systems [2] are major limitations in HBV research which restrain the study of this major human pathogen. But the establishment of the animal model with domestic duck employing the DHBV has helped greatly to overcome the shortcomings in HBV research [1,3]. However, this model has its own limitations as revealed by the differences in the clinical manifestations of the disease in humans and birds infected by these viruses. This mainly pertains to the chronicity in DHBV infection without liver injury/hepatocellular carcinoma (HCC)/cirrhosis; spontaneous elimination of infection in adult ducks; and at the molecular level, the expression of only a cryptic X-protein [4]. A major lacuna in HBV biology is the lack of sufficient information on the molecular mechanisms involved in the development of HCC in chronic HBV patients, which has become a major medical challenge [5].

A few studies have been performed comparing the gene expression in HBV positive HCC and non-cancerous liver [6] and viral and non-viral HCC [7] in patient samples. However, no study has focused to identify the differential gene expression in infection with DHBV either *in vivo *or *in vitro *to facilitate a comparative analysis. A recent *in vitro *study has addressed the proteomic changes during DHBV infection, which has brought to light a number of genes that are involved in the infection process [8]. However, a purely proteome based approach might not reveal changes in the expression levels of many of the low abundant proteins due to technical limitations, which needs to be complemented by mRNA/cDNA differential expression based approaches. In this context, we carried out a subtractive hybridization cDNA library construction and screening to identify the differential gene expression during DHBV infection in primary duck hepatocytes (PDH) in culture. The protocol we followed identified 42 up-regulated and 36 down-regulated genes in DHBV infected PDH in culture.

## Methods

Primary duck hepatocytes (PDH) were isolated from 27-day old embryonated, un-hatched, duck eggs free of duck hepatitis B virus (DHBV) infection as previously described [9] and maintained at 5 × 10^6 ^cells/ml in DMEM+F12 (Sigma) and 5% FBS supplemented with glucose (0.5 gm/l), dexamethasone (10^-5 ^M) and insulin (1 μg/ml) (all from Sigma) at 37°C in a 5% CO_2 _atmosphere. DHBV stock was concentrated from LMH-D2 cell culture supernatant, a chicken hepatoma cell line that constitutively replicate DHBV, (a kind gift from Dr. William S Mason, Fox Chase Cancer Centre, California), by precipitation with 10% polyethylene glycol 8000 (USB, USA) [10]. The pellet was re-suspended in DMEM+F12 medium and this concentrated virus was used to infect PDH at an MOI of 10^3 ^genome equivalents per hepatocyte, as previously described [11] in presence of 1% DMSO (Sigma). DHBV infection was confirmed by PCR on the DNA obtained from the culture supernatant using DHBV specific primers P1F and D2R (Additional File 1, Table 1).

**Table 1:** List of cDNAs up-regulated during PDH infection with DHBV

**No**.	Name of the clone	Abundance Ratio	BLAST Result	BLAST/tBLASTx	Amplicon Size (bp)	e-value	**GenBank Accession No**.
1	F22	2.41	Cadherin 11, type 2, OB-cadherin (osteoblast) (CDH11)-Gallus gallus	BLAST	462	0	JG662697
2	F125	2.23	Pyruvate dehydrogenase E1-beta subunit variant 3-like-Taeniopygia guttata	BLAST	268	2.00E-85	JG662698
3	F106	2.08	Anas platyrhynchos female-specific sequence	tBLASTx	371	3.00E-04	JG662699
4	F8	2.04	Similar to SH3 domain containing 19-Taeniopygia guttata	tBLASTx	678	4.00E-07	JG662700
5	F71	2	Ubiquitin-like, containing PHD and RING finger domains, 1 (UHRF1)-Gallus gallus	BLAST	593	0	JG662701
6	F21	1.87	Zinc finger CCCH-type containing 13 (ZC3H13)-Gallus gallus	BLAST	681	0	JG662702
7	F13	1.77	Succinate-CoA ligase, GDP-forming, alpha subunit(SUCLG1)-Gallus gallus	BLAST	767	0	JG662703
8	F76	1.76	ElaC homolog 2 (E. coli) (ELAC2)-Gallus gallus	tBLASTx	218	1.00E-14	JG662704
9	F19	1.58	CWC22 spliceosome-associated protein homolog-Taeniopygia guttata	BLAST	680	0	JG662705
10	F70	1.55	Similar to KIAA2019 protein/AHNAK nucleoprotein 2-Gallus gallus	BLAST	544	1.00E-150	JG662706
11	F46	1.51	Filamin B, beta-Gallus gallus	BLAST	655	1.00E-140	JG662707
12	F77	1.45	Tumor necrosis factor receptor superfamily,member 6b, decoy (TNFRSF6B)-Gallus gallus	tBLASTx	527	5.00E-07	JG662708
13	F131	1.44	Nuclear protein Matrin 3 (MATR3)-Gallus gallus	BLAST	448	0	JG662709
14	F45	1.42	Heat shock transcription factor 2 (HSF2)-Gallus gallus	BLAST	755	0	JG662710
15	F74	1.42	High mobility group AT-hook 2 (HMGA2)-Gallus gallus	BLAST	209	2.00E-49	JG662711
16	F6	1.4	CLE7-Gallus gallus	BLAST	541	0	JG662712
17	F16	1.35	Cu/Zn superoxide dismutase (SOD1)-Melopsittacus undulatus	BLAST	346	8.00E-31	JG662713
18	F43	1.33	Exonuclease NEF-sp-Gallus gallus	BLAST	680	9.00E-143	JG662714
19	F26	1.32	Component of oligomeric golgi complex 3 (COG3)-Bos taurus	tBLASTx	308	0.002	JG662715
20	F42	1.31	CD9 protein-Anas platyrhynchos	BLAST	748	0	JG662716
21	F127	1.31	Junco hyemalis 164 gene, partial sequence	tBLASTx	234	5.00E-16	JG662717
22	F17	1.29	Quaking homolog, KH domain-Taeniopygia guttata	BLAST	678	0	JG662718
23	F44	1.29	Alanine-glyoxylate aminotransferase 2-Gallus gallus	tBLASTx	721	5.00E-21	JG662719
24	F135	1.27	Leucine-rich repeats and calponin homology (CH) domain containing 4-Oryctolagus cuniculus	tBLASTx	288	0.4	JG662720
25	F14	1.24	Clathrin, light chain A (CLTA)-Gallus gallus	BLAST	673	0	JG662721
26	F83	1.2	Sequestosome 1-Gallus gallus	BLAST	562	0	JG662722
27	F10	1.18	RAB 32, member of Ras oncogene-Gallus gallus	BLAST	743	0	JG662723
28	F30	1.16	Ribosomal protein L6 (RPL6)-Gallus gallus	BLAST	591	0	JG662724
29	F64	1.16	Holocytochrome c synthase (cytochrome c heme-lyase)-Gallus gallus	tBLASTx	421	8.00E-52	JG662725
30	F32	1.16	Lysosomal-associated membrane protein 1-Taeniopygia guttata	tBLASTx	591	4.00E-93	JG662726
31	F7	1.14	Serine protease 23-Gallus gallus	BLAST	740	3.00E-168	JG662727
32	F18	1.09	Beta-catenin isolate 3-Anas platyrhynchos	BLAST	710	0	JG662728
33	F52	1.09	Zebrafish DNA sequence from clone CH211-276C22 in linkage group 6	tBLASTx	218	2.2	JG662729
34	F25	1.08	Gallus gallus finished cDNA, clone ChEST457d18	tBLASTx	696	2.00E-27	JG662730
35	F59	1.07	Leucine proline-enriched proteoglycan (leprecan)1/prolyl 3-hydroxylase 1 (P3H1)-Gallus gallus	BLAST	581	0	JG662731
36	F12	1.07	Ribophorin I-Gallus gallus	BLAST	796	0	JG662732
37	F87	1.06	Gallus gallus finished cDNA, clone ChEST855m19	BLAST	505	3.00E-91	JG662733
38	F95	1.06	Spastic paraplegia 3A (autosomal dominant)-Gallus gallus	BLAST	316	3.00E-54	JG662734
39	F107	1.06	High-mobility group box 3-Taeniopygia guttata	BLAST	276	2.00E-136	JG662735
40	F1	1.05	ATP synthase, H+ transporting, mitochondrial F0 complex, subunit F2 (ATP5J2)-Gallus gallus	tBLASTx	199	9.00E-18	JG662736
41	F78	1.03	Ubiquitin specific peptidase 47 (USP47)-Gallus gallus	BLAST	695	0	JG662737
42	F88	1.03	No significant similarity found	tBLASTx	360	-	JG662738

2 μg of polyA RNA each from DHBV infected and uninfected PDH on zero and 4^th ^day of infection was isolated using PolyATract mRNA isolation system-III (Promega, USA) and was used to construct forward and reverse subtracted cDNA libraries using Clontech PCR-Select cDNA subtraction kit (Clontech, USA), as per kit protocols. PCR amplification of a house-keeping gene GAPDH (Additional File 1, Table 1) from subtracted and un-subtracted samples was used for confirmation of the subtraction efficiency. The subtracted cDNAs were ligated with the pGEM-T (Easy) vector (Promega), competent JM109 *Escherichia coli *cells (Promega) were transformed and plasmids were isolated following standard molecular biology protocols to obtain 137 forward and 148 reverse subtracted clones.

Macroarrays of these plasmids were generated by vacuum transferring 100 ng each of the denatured clone plasmid in duplicate spots onto nylon membranes (Hybond-N+, Amersham Biosciences UK) using a dot-blot apparatus (Bio-Dot, Bio-Rad). The arrays were hybridized with α^32 ^P labelled forward and reverse subtracted cDNA mixtures as radioactive probes in a reverse-northern procedure. The probes were radio-labelled in a 50 μl PCR reaction using [α^32 ^P]-dCTP, dATP, dGTP, dTTP (0.2 mM each) and unlabelled dCTP (0.02 mM) using the nested PCR primers 1 and 2R (10 μM each) (Additional File 1, Table 1) and the Advantage 2 polymerase mix (Clontech). The adaptor regions common to both the probe and library clones were removed by digestion with RsaI restriction enzyme (NEB). The arrays were individually hybridized with both forward and reverse radio-labelled probes. Subsequent to a pre-hybridization of the membrane for 30 min in the hybridization solution(10% Polyethylene glycol, 1.5× SSPE and 7% sodium dodecyl sulphate), heat denatured probe solution containing 100 μl of RsaI digested radio-labelled probe, 250 μl of 10 mg/ml Herring sperm DNA(Promega) and 100 μl of 0.2N NaOH was added. The probe solution was removed after 16 hrs of hybridization at 65°C and the membrane was washed twice in 2× SSC and 0.1%SDS for 10 min at room temperature followed by two high stringency washes using 0.2× SSC and 0.1%SDS at 65°C for 10 min, and exposure to a phosphor screen for 30 min. The images were captured in Molecular Imager FX (Bio-Rad). The hybridization intensity was measured in the captured images by densitometry analysis of the signal on individual clones using VisionWorksLS image acquisition and analysis software (UVP, USA). The relative abundance ratio of gene expression was calculated using the following formulas.

All genes with an abundance ratio of more than one, a cut-off fixed arbitrarily, were then short-listed as the ones with true differential expression. These clones were subjected to automated DNA sequencing in an ABI Prism 310 sequencer (Applied Biosystems) with the Big Dye Terminator 3.0 kit (ABI Prism; Applied Biosystems) as per the manufacturer's directions using the primers TvectF and TvectR (Additional File 1, Table 1). The sequences thus obtained were analyzed using the BLAST online software (NCBI).

Three genes, randomly selected from the top five genes in Table 1 and 2 (with high abundance); one gene from the bottom (with lower abundance) of the table; and one gene, which was not short-listed, were used for real-time PCR analysis for validation of the short-listing procedure. Specific primers for these 10 genes (five from each of the up-regulated and down-regulated library) and primers for the house keeping gene GAPDH were designed (Additional File 1, Table 1) and used in the real-time PCR. cDNA was synthesized using total RNA from fresh sets of primary duck hepatocyte cultures either infected with DHBV or uninfected, as described above, using Avian Myeloblastosis Virus (AMV) reverse transcription system (Promega). Real-time PCR was carried out as previously described [12]. The experiments were repeated thrice, each in duplicates, and average fold change in gene expression was calculated for individual genes.

**Table 2:** List of cDNAs down-regulated during PDH infection with DHBV

**No**.	Name of the clone	Abundance Ratio	BLAST Result	BLAST/ tBLASTx	Amplicon Size (bp)	e-value	**GenBank Accession No**.
1	R73	1.52	Ferritin, heavy polypeptide 1 (FTH1)-Gallus gallus	BLAST	342	4.00E-143	JG662661
2	R90	1.41	Zinc finger CCCH-type, antiviral 1 (ZC3HAV1)-Gallus gallus	tBLASTx	328	1.00E-14	JG662662
3	R130	1.39	T-complex 1-Taeniopygia guttata	BLAST	400	6.00E-172	JG662663
4	R108	1.39	Y box binding protein 1-Gallus gallus	BLAST	486	0	JG662664
5	R96	1.34	MYST/Esa1-associated factor 6-Taeniopygia guttata	BLAST	646	1.00E-17	JG662665
6	R97	1.33	Similar to RGD-CAP-Gallus gallus	BLAST	743	0	JG662666
7	R111	1.31	PREDICTED: Gallus gallus similar to Ankycorbin	BLAST	646	0	JG662667
8	R134	1.3	No significant similarity found	tBLASTx	490	-	JG662668
9	R123	1.25	ATPase8, ATPase6 genes for F0-ATP synthase subunit 8, F0-ATP synthase subunit 6-Anas platyrhynchos	BLAST	462	0	JG662669
10	R133	1.24	Versican-Gallus gallus	BLAST	257	3.00E-98	JG662670
11	R103	1.22	Platelet-activating factor acetylhydrolase isoform Ib, alpha subunit 45kDa (PAFAH1B1)-Gallus gallus	BLAST	508	4.00E-145	JG662671
12	R16	1.18	UPF0308 protein-Gallus gallus	BLAST	593	0	JG662672
13	R100	1.17	TRAF interacting protein (TRAIP)-Gallus gallus	BLAST	631	0	JG662673
14	R84	1.16	Acid alpha-glucosidase-Macaca mulatta	tBLASTx	756	3.8	JG662674
15	R15	1.15	Microtubule-associated protein RP/EB family, member 1-Taeniopygia guttata	BLAST	438	5.00E-168	JG662675
16	R126	1.15	Catechol-O-methyltransferase-Gallus gallus	tBLASTx	395	1.00E-25	JG662676
17	R135	1.14	Chromosome 15 hypothetical ATG/GTP binding protein-Gallus gallus	tBLASTx	239	0.048	JG662677
18	R143	1.13	Ankyrin repeat domain 17 (ANKRD17)-Gallus gallus	BLAST	546	0	JG662678
19	R45	1.13	Splicing factor, arginine/serine-rich 18 (SFRS18)-Gallus gallus	tBLASTx	476	1.00E-145	JG662679
20	R141	1.11	Cytochrome oxidase subunit I (COI)-Anas platyrhynchos	BLAST	336	1.00E-152	JG662680
21	R10	1.11	Eukaryotic translation initiation factor 5 (EIF5)-Gallus gallus	BLAST	735	0	JG662681
22	R129	1.11	Beta-actin-Anas platyrhynchos	BLAST	664	0	JG662682
23	R106	1.1	Alpha enolase-Peking Duck	BLAST	381	0	JG662683
24	R93	1.1	No significant similarity found	tBLASTx	488	-	JG662684
25	R139	1.1	Similar to KIAA1824 protein/WD repeat domain 22-Gallus gallus	BLAST	279	7.00E-100	JG662685
26	R104	1.09	Caspase 3, apoptosis-related cysteine peptidase (CASP3)-Gallus gallus	BLAST	792	0	JG662686
27	R95	1.09	Gallus gallus finished cDNA, clone ChEST757h13	tBLASTx	793	5.00E-19	JG662687
28	R79	1.07	Ral guanine nucleotide dissociation stimulator-like 1 (RGL1)-Gallus gallus	BLAST	333	1.00E-132	JG662688
29	R105	1.06	Gallus gallus similar to MGC53471 protein	BLAST	646	7.00E-89	JG662689
30	R128	1.05	Hydroxyacyl glutathione hydrolase-like,transcript variant 2-Taeniopygia guttata	BLAST	382	4.00E-54	JG662690
31	R22	1.05	Proteasome (prosome, macropain) 26S subunit, ATPase,1 (PSMC1)-Gallus gallus	BLAST	324	4.00E-133	JG662691
32	R99	1.04	Gallus gallus hypothetical protein	BLAST	362	2.00E-86	JG662692
33	R2	1.04	Ribosomal protein L35a-Gallus gallus	BLAST	90	3.00E-08	JG662693
34	R36	1.04	Gallus gallus finished cDNA, clone ChEST191i5	tBLASTx	414	0.025	JG662694
35	R124	1.02	Gallus gallus BAC clone CH261-189F16 from chromosome z	BLAST	524	0	JG662695
36	R86	1.02	Transmembrane protein 30A-Taeniopygia guttata	BLAST	220	4.00E-72	JG662696

The threshold cycle (C_t_) values obtained in the real-time PCR analysis were normalized with the expression of the house-keeping gene GAPDH, and the relative expression of individual genes in infected and uninfected cells were calculated by Pfaffl method [13] for Day 0 and Day 4 of infection using the equation:

The ratios for day 0 and day 4 infected samples were compared and analysed statistically by paired Student's t-test to validate the significance of gene expression changes. P-values < 0.05 were considered significant.

## Results & Discussion

**Figure 1:**
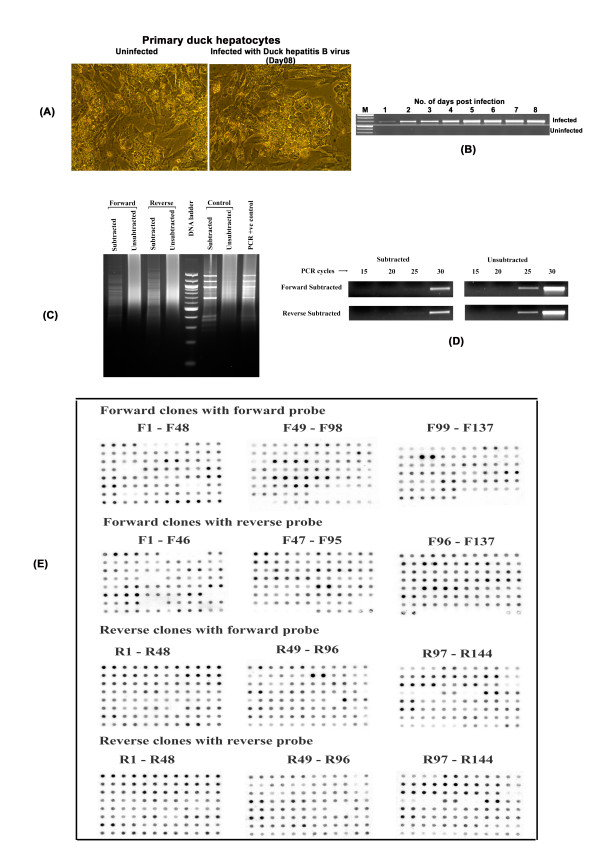
**Subtractive hybridization cDNA library construction and screening**. (A) PDH infected with DHBV, 8 days post-infection. (B) PCR Confirmation of DHBV infection. Upper lane shows the increase in amplification of a DHBV specific gene from days 1 through 8, while the amplification is missing from uninfected controls. (C) Comparison of subtracted and unsubtracted cDNAs on a 2% agarose gel. Individual lanes are marked. Lane 5 is a 100 bp DNA ladder. Lane 8 is a positive control provided with the kit. (D) Analysis of subtraction efficiency using PCR for GAPDH. (E) Macroarray screening by dot-blot hybridization. Each clone is spotted in duplicates. Membranes were hybridized with radio-labelled probes as indicated. The average densitometric intensities of each duplicate clone pair was read for relative abundance calculation.

**Figure 2:**
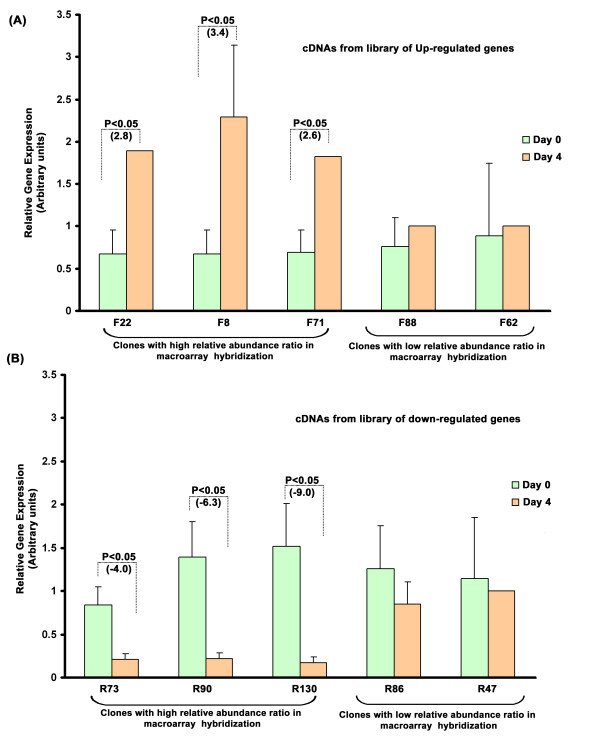
**Real-time PCR of representative genes in DHBV infected PDH, 0-day and 4-days post-infection**. (A) Significant up-regulation of cDNAs (F22, F8, and F71) selected from the top of short-listed clones in the up-regulated gene table (Table-1). (B) Significant down-regulation of cDNAs (R73, R90, and R130) selected from the top of short-listed clones in the down-regulated gene table (Table-2). The Y-axis represents relative gene expression values obtained from the Pfaffl analysis (see Methods). Significant P-values (< 0.05) are indicated. The values in parenthesis indicate fold-change in expression.

The infection of PDH with DHBV did not produce any visible changes on the cell monolayer (Figure 1A). The virus infection was confirmed by PCR detection of a 300 bp DHBV glycoprotein 1 (gp1) gene fragment in the DNA isolated from infected PDH culture supernatant (Figure 1B) and by sequence analysis. The establishment of a productive infection was indicated by the increasing PCR amplification intensity of the gene fragment with every successive day of culture for the total culture period of eight days. For RNA isolation for subtraction library construction, we selected an early time point of 4 days as described in previous studies [14]. Two libraries were generated- the forward subtracted or up-regulated genes and the reverse subtracted or down-regulated genes. The efficiency of subtraction procedure was indicated by a decrease in the intensity and appearance of discrete banding patterns in the lanes with subtracted products (Figure 1C) and was confirmed by PCR detection of the house-keeping gene GAPDH, the amplicons of which appeared at an earlier time point (25 cycles) in un-subtracted samples compared to a later time point (30 cycles) in both forward and reverse subtracted libraries (Figure 1D). Hybridization of macroarrays blotted with 137 up-regulated and 148 down-regulated clones (Figure 1E) and short-listing only the ones with an abundance ratio of more than 1, we obtained 42 non-redundant up-regulated clones and 36 non-redundant down-regulated clones (Tables 1 and 2). Real-time PCR done using the representative sets of short-listed clones gave results confirming the reliability of the short-listing procedure. Genes that topped the differential expression among the up-regulated genes (F22, F8, F71) showed a significant (P < 0.05) increase in expression at 4-days compared to the 0 day in infected PDH (Figure 2A), while the reverse was the case of the down-regulated genes (R73, R90, R130) (Figure 2B), all of whose expression decreased significantly (P < 0.05) at 4-day DHBV infection. F88 and R86, which were selected from the bottom end of the up-regulated and down-regulated gene-tables, respectively, also showed the expected modulation albeit at a lower fold. F62 and R47, picked from the genes left-out did not show any significant difference in their expression pattern.

Functional classification of the short-listed clones using gene ontology based on BLAST results grouped them mainly into those belonging to cellular processes such as cellular respiration, signal transduction, transcription/translation, ubiquitin/proteasome pathway and apoptosis besides those coding for membrane and cytoskeletal proteins (Table 3). Among them, the category that was maximum up-regulated were the ones involved in transcription/translation (19%), whereas the ones maximum down regulated (11%) belonged to cytoskeletal proteins. The former included the HMG Box proteins and Y-box binding proteins. Previous studies have implicated the Y-box binding protein1, Platelet-activating factor acetylhydrolase isoform 1B (PAFAH1B1), Ribosomal Protein L35a, Ferritin, α-enolase, Caspase 3, CuZn Superoxide Dismutase (CuZnSOD), Filamin B, Pyruvate dehydrogenase 1-β, β-catenin, prolyl-3-hydroxylase 1, β-actin, acid α-glucosidase, and clathrin, the cDNAs of which were identified to be up-regulated, with chronic HBV infections and HCC development [6,15-26]. In comparison to the earlier report based on proteome analysis in DHBV infected PDH [8], except for β-actin and α-enolase, all the cDNAs identified in the present study represented new genes. The difference could be due to multiple reasons, and importantly it might include the selective enrichment/elimination of some of the cDNAs during the process of RT-PCR amplification and cloning as part of the subtraction library construction. Nevertheless, our data provides a new set of candidate genes worth further investigation in hepadnaviral infection.

**Table 3:** Categorization of genes according to the reported function available from literature

	FORWARD	REVERSE
**Membrane proteins**	Cadherin 11	Transmembrane protein 30A
	Lysosomal-associated membrane protein 1	
	CD9 protein	
	Leucine-rich repeats and calponin homology (CH) domain containing 4	

**Cellular Respiration**	Pyruvate dehydrogenase E1-beta subunit	Alpha enolase
	Succinate-CoA ligase, GDP-forming, alpha subunit(SUCLG1)	Hydroxyacyl glutathione hydrolase-like
	ATP synthase, H+ transporting, mitochondrial F0 complex, subunit F2(ATP5J2)	ATPase8, ATPase6 genes for F0-ATP synthase subunit 8, F0-ATP synthase subunit 6
		Cytochrome oxidase subunit I (COI)

**Cytoskeletal**	Filamin B, beta	Beta-actin
		T-complex 1
		Microtubule-associated protein RP/EB family, member 1
		Similar to Ankycorbin

**Signal Transduction**	Beta-catenin isolate 3	TRAF interacting protein (TRAIP)
	Quaking homolog, KH domain	

**Transcription and Translation**	High mobility group AT-hook 2 (HMGA2)	Splicing factor, arginine/serine-rich 18 (SFRS18)
	High-mobility group box 3	MYST/Esa1-associated factor 6
	Heat shock transcription factor 2 (HSF2)	Y box binding protein 1
	CWC22 spliceosome-associated protein homolog	Ribosomal protein L35a
	Ubiquitin-like, containing PHD and RING finger domains, 1 (UHRF1)	Eukaryotic translation initiation factor 5 (EIF5)
	Nuclear protein Matrin 3 (MATR3)	
	Ribosomal protein L6 (RPL6)	
	Ribophorin I	

**Ubiquitin-proteasome**	Ubiquitin specific peptidase 47 (USP47)	Proteasome (prosome, macropain) 26S subunit, ATPase,1 (PSMC1)
	Sequestosome 1	

**Apoptosis**	Tumor necrosis factor receptor superfamily, member 6b, decoy (TNFRSF6B)	Caspase 3, apoptosis-related cysteine peptidase (CASP3)

**Others**	Anas platyrhynchos female-specific sequence	No significant similarity found
	Junco hyemalis 164 gene, partial sequence	UPF0308 protein
	Gallus gallus finished cDNA, clone ChEST457d18	No significant similarity found
	No significant similarity found	Gallus gallus finished cDNA, clone ChEST757h13
	Gallus gallus finished cDNA, clone ChEST855m19	Gallus gallus finished cDNA, clone ChEST191i5
	Zebrafish DNA sequence from clone CH211-276C22 in linkage group 6	Gallus gallus BAC clone CH261-189F16 from chromosome z
	Similar to SH3 domain containing 19	Gallus gallus hypothetical protein
	Zinc finger CCCH-type containing 13 (ZC3H13)	Gallus gallus similar to MGC53471 protein
	ElaC homolog 2 (E. coli) (ELAC2)	Ferritin, heavy polypeptide 1 (FTH1)
	Similar to KIAA2019 protein/AHNAK nucleoprotein 2	Zinc finger CCCH-type, antiviral 1 (ZC3HAV1)
	CLE7	Similar to RGD-CAP
	Cu/Zn superoxide dismutase (SOD1)	Versican
	Exonuclease NEF-sp	Platelet-activating factor acetylhydrolase isoform Ib, alpha subunit 45kDa (PAFAH1B1)
	Component of oligomeric golgi complex 3 (COG3)	Catechol-O-methyltransferase
	Clathrin, light chain A (CLTA)	Chromosome 15 hypothetical ATG/GTP binding protein
	RAB 32, member of Ras oncogene	Ankyrin repeat domain 17 (ANKRD17)
	Holocytochrome c synthase (cytochrome c heme-lyase)	Similar to KIAA1824 protein/WD repeat domain 22
	Serine protease 23	Ral guanine nucleotide dissociation stimulator-like 1 (RGL1)
	Leucine proline-enriched proteoglycan (leprecan)1/prolyl 3-hydroxylase 1 (P3H1)	Acid alpha-glucosidase
	Spastic paraplegia 3A (autosomal dominant)	
	Alanine-glyoxylate aminotransferase 2	

An interesting observation in this study was the inverse pattern of differential expression of ten of these genes in *in vitro *DHBV infected cells as against the reports on HCC clinical samples [6,15-20]. The mRNAs for the Y-box binding protein1, PAFAH1B1, Ribosomal Protein L35a, Ferritin, α-enolase, acid alpha-glucosidase and Caspase 3 were shown to be down-regulated during *in vitro *DHBV infection, whereas those of CuZnSOD, Filamin B and Pyruvate dehydrogenase were shown to be up-regulated, where as the reverse was the trend in human HCC. This observation may be purely coincidental owing to the fact that the experimental method we used was an *in vitro *system, and the changes in primary hapatocytes during culture itself, such as de-differentiation, might have led to these alterations in gene expression.

## Conclusions

In summary, the present study identified cDNAs of a number of genes that are differentially modulated in cultured PDH, *invitro *infected with DHBV. cDNAs of both novel as well as already reported genes/proteins associated with HBV/DHBV infection or HCC were identified in the library. The genes short-listed here could be valuable leads for further studies in animal models, which might help to understand the pathology of chronic HBV infections and pathogenesis of HCC.

## Competing interests

The authors declare that they have no competing interests.

## Authors' contributions

SN, DSA and AI carried out the experiments. SN drafted the manuscript. ES conceived the study, edited and completed the final version of the manuscript. All authors read and approved the final manuscript.

## Supplementary Material

Additional file 1 **Primers used in the study**. Click here for file
