# Supplementary material for: Differential gene expression analysis of in vitro duck hepatitis B virus infected primary duck hepatocyte cultures
Source: Virol J. 2011 Jul 23;8:363. doi: 10.1186/1743-422X-8-363 (PMC3152538; doi:10.1186/1743-422X-8-363)
Supplement: Additional file 1 — Primers used in the study. [file 1743-422X-8-363-S1.PDF]

Additional File 1 - Primers used in the study

	Target	Primer name	Sequence (5` to 3`)	Amplicon Size (bp)	Ta(°C)
**Primers used for DHBV detection**					
	**Target**	**Primer name**	**Sequence (5` to 3`)**	**Amplicon Size (bp)**	**Ta(°C)**
	Glycoprotein 1 (gp1)	DHBV-P1F	AATGAGGAATCATTGGATAGGGC	300	60
		DHBV-D2R	CCTTTCCCTGGACTTTGAACG		60
**Primers used in screening subtraction library**					
	**Target**	**Primer name**	**Sequence (5` to 3`)**	**Amplicon Size (bp)**	**Ta(°C)**
	Adaptor region flanking clones	Nested PCR primer 1	TCGAGCGGCCGCCCGGGCAGGT	Varies depending on insert	68
		Nested PCR primer 2R	AGCGTGGTCGCGGCCGAGGT		68
**List of primers used Real-time PCR amplication of Up-regulated (Forward) clones**					
	**Target**	**Primer name**	**Sequence (5` to 3`)**	**Amplicon Size (bp)**	**Ta(°C)**
	Clone F22	F22 F	TCGAGTCTGCGGCTGCGACA	103	63
		F22 R	GAGAATGGCGATTAAAGCTCCCGT		63
	Clone F8	F8 F	GCGCAGACAACTCCACCCCCAGAA	101	63
		F8 R	GCAAGCAAGCATCATCCGCTTTGG		63
	Clone F71	F71 F	GGCTCTGAACTGTAGTGCCCCCA	89	63
		F71 R	TCCTCACCACCCGGACTGGCTT		63
	Clone F88	F88 F	GTCAACCAGAAGTCGCTTTCTGCA	106	59
		F88 R	CAGGCACTGTAGCCAATGCAC		59
	Clone F62	F62 F	TTAGCAGGCCCAGCTGTTGACG	102	59
		F62 R	ACAGCTTGCTGATGAGCTGCT		59

List of primers used Real-time PCR amplication of Down-regulated (Reverse) clones					
	**Target**	**Primer name**	**Sequence (5` to 3`)**	**Amplicon Size (bp)**	**Ta(°C)**
	Clone R73	R73 F	ACCAGTCGCTGTTGGAGCTGCACA	139	63
		R73 R	TTCCGCAGGTTGGTGACATGGTCG		63
	Clone R90	R90 F	TGCACTGTGTGCAACAAAACGCA	187	59
		R90 R	TGCTTAGCAAATGCTGTAGGACCA		59
	Clone R130	R130 F	AACAACCTGCACACCGAGCTTCAT	152	63
		R130 R	GCAGCCAAAAAGAGAGCATTCTCG		63
	Clone R86	R86 F	CAAGTGGCCTGCCCAGTTAG	90	58
		R86 R	GACGGGTATACAATTCGCTCCA		58
	Clone R47	R47 F	TGCAAGGCTTCATACAGGCAAC	111	58
		R47 R	TAGGCCAAAGCCAGACAGTG		58
**Primers used for Real-time PCR normalization and checking subtraction efficiency**					
	**Target**	**Primer name**	**Sequence (5` to 3`)**	**Amplicon Size (bp)**	**Ta(°C)**
	Glyceraldehyde 3 phosphate dehydrogenase	D-GAPDH F	ACTCATGGCCACTTCCGGGGCA	196	63
		D-GAPDH-R	TAGCACCACCCTTCAGGTGAGCA		63
